# High frequency of methicillin-resistant *Staphylococcus aureus* in Peshawar Region of Pakistan

**DOI:** 10.1186/s40064-016-2277-3

**Published:** 2016-05-11

**Authors:** Asad Ullah, Muhammad Qasim, Hazir Rahman, Jafar Khan, Mohammad Haroon, Niaz Muhammad, Abdullah Khan, Noor Muhammad

**Affiliations:** Department of Microbiology, Kohat University of Science and Technology, Kohat, Khyber Pakhtunkhwa 26000 Pakistan; Medical ICU Unit, Khyber Teaching Hospital, Peshawar, Khyber Pakhtunkhwa Pakistan; Department of Biotechnology and Genetic Engineering, Kohat University of Science and Technology, Kohat, Khyber Pakhtunkhwa Pakistan

**Keywords:** Epidemiology, Drug resistance, *S. aureus*, MRSA, Peshawar

## Abstract

*Staphylococcus aureus* is an increasing problem in clinical practice because of reduced susceptibility to available antibiotics. The objective of the study was to determine the frequency of Methicillin-resistant *S.aureus* (MRSA) in Peshawar, Pakistan. Clinical isolates of *S. aureus* were subjected to determination of antibiotic resistance, MICs and inducible clindamycin resistance (ICR). Out of total 280 *S. aureus* isolates, the frequency of MRSA was 36.1 % (n = 101). MRSA infection was found higher among the age group 50–59 years (60.71 %, OR 3.09), followed by 20–29 years (47.5 %, OR 1.74). Frequency of MRSA in female and male was 39.8 and 34 % respectively. MRSA was more frequent in blood specimens (48.7 %, OR 2.14). The frequency of community and hospital acquired MRSA was 42 and 34.8 % respectively. MRSA showed high resistance (100 %) to penicillin and cefoxitin followed by erythromycin (99 %). While MRSA exhibited 100 % susceptibility to vancomycin and linezolid. We have also found 7 vancomycin intermediate sensitive *S. aureus* (VISA) isolates. ICR was observed in 15.84 % (n = 16) of MRSA isolates. It is concluded that MRSA is potential threat to public health in Peshawar. Vancomycin and linezolid could be prescribed as a drug of choice in treating MRSA associated infections. In addition, ICR should be routinely checked to avoid clindamycin treatment failure.

## Background

*Staphylococcus aureus* (*S. aureus*) is a serious risk to public health as it causes human infections ranges from wound abscess to life threatening conditions (Deleo et al. 2010). Emergence of antibiotics resistant *S. aureus* especially Methicillin-resistant *Staphylococcus aureus* (MRSA) is a worldwide problem in both healthcare and community settings (Chambers and DeLeo 2009). MRSA can be transmitted from person to person via skin or the sharing of contaminated objects. In addition, MRSA can evade host immune system and virulence factors disseminated render this bug with limited therapeutic options available (Holcomb et al. 2008). MRSA have capability to breed in the presence of methyl penicillin and its derivatives like methicillin. Resistance to methicillin is mediated by *mec*-*A* gene, which encodes the polypeptide PBP2a protein (Oliveira et al. 2006). The *mec*-*A* gene have also insertion sites for transposons and plasmids which assist resistance to other antibiotic groups. Accordingly, cross-resistance to non-beta-lactam antibiotic groups including quinolones, sulfamethoxazole, macrolides, aminoglycoside and lincomycin were frequently observed in MRSA isolates (Chambers 2001).

It has been reported that the MRSA infections are not limited to human but it can also infect domestic or farm animals such as horses, sheep, goat, and cattle (Saleha and Zunita 2010). MRSA has worldwide distribution but their frequency varies among various countries. The frequency of MRSA in Pakistan and India has been shown to be high as compared to northern Europe (Anwar et al. 2004). In Pakistan, the prevalence of methicillin resistance in *S. aureus* has been observed with range from 42 to 51 % (Akinkunmi and Lamikanra 2012). With the increase of MRSA associated infections, the use of glycopeptides (vancomycin and teicoplanin) are also rising gradually. The irrational and indiscriminate use of glycopeptides results in the emergence of even low-level vancomycin resistance in *S. aureus.* Such resistance, though rare, but it is an emerging threat because of its potential to disseminate rapidly (Perwaiz et al. 2007). The present study was designed to investigate the frequency of MRSA in Peshawar. Findings of the study will be helpful for devising an appropriate hospital antibiotic stewardship policy to reduce the chances of *S. aureus* associated infections in the area.

## Methods

### Ethical considerations

The current study was approved by departmental research ethics committee (DREC) of the Department of Microbiology, Kohat University of Science and Technology, Kohat, Pakistan.

### Samples description

The present study was carried out at Peshawar, the provincial capital of Khyber Pakhtunkhwa, Pakistan with confluence of local and Afghan refugee’s population. Clinical samples received at Pathology Department of Rehman Medical and Teaching Institute Peshawar, Pakistan from September 2012 to September 2013 were included in the study. An informed written consent was taken from patients to collect data regarding patient’s history, age and sex. In the present study different clinical samples were randomly collected from patients who visited or admitted in the hospital during study duration. MRSA Infection origin (hospital acquired vs community acquired) was also determined. Hospital acquired infection is an infection that develops in the hospital and was not present at the time of admission while a community-acquired infection has been defined as an infection that was present at the time of admission in the hospital. This definition is as per CDC definition (Garner et al. 1988; Salgado et al. 2003).

### Culture and biochemical identification of *S. aureus*

Randomly collected clinical samples such as pus, fluid, blood, tissue and throat were processed for bacteria culturing followed by *S. aureus* identification based on Gram’s staining, culture characteristics and biochemical reactions such as Catalase, Coagulase and DNase (Perwaiz et al. 2007). A total of 280 samples which were positive for *S. aureus* were further processed for antibiotic susceptibility to screen MRSA and MSSA.

### Phenotypic based detection of MRSA

All confirmed *S. aureus* isolates were screened for methicillin resistance using cefoxitin (Fox) disc and oxacillin resistant screening agar base medium (ORSB) method as described (Velasco et al. 2005). Briefly, bacterial colony was picked up from overnight culture and inoculated into ORSAB medium followed by incubation at 35 °C for 24 to 48 h. Presence of growth on ORSB was indicative of Oxacillin resistance. To check FOX resistance, bacterial lawn was prepared on Mueller–Hinton agar and FOX disk was placed on it, followed by incubation at 37 °C for overnight. Growth around the FOX disk was indicative of FOX resistance.

### Molecular based detection of MRSA

Total DNA was extracted using Nucleospin^®^ tissue genomic DNA kit according to the vendor protocol (Macherey–Nagel, Germany). The purified DNA was subjected to PCR based amplification of *mecA* gene using specific primers (Forward: 5′-AAAATCGATGGTAAAGGTTGGC-3′; Reverse 5′-AGTTCTGCAGTACCGGATTTGC-3′) as described earlier (Lee 2003). The amplified product was resolved on agarose gel electrophoresis and then visualized under UV transilluminator.

### Antibiotic susceptibility assay

Antibiotic susceptibility test was carried out by disc diffusion method (Velasco et al. 2005). Bacterial overnight culture matched with 0.5 McFarland standards was inoculated on Mueller–Hinton agar plates and various antibiotic discs were placed followed by incubation at 37 °C for 24 h. Afterward, an antibiotic zone of inhibition was measured and interpreted as per the CLSI guidelines (CLSI 2012).

### MIC determination of vancomycin against MRSA isolates by E-test method

The MIC to vancomycin was performed by E-test method (Oxoid, England) and interpreted as per the CLSI guidelines (CLSI 2012).

### Detection of inducible clindamycin resistance by D-test

Inducible clindamycin resistance for MRSA isolates was checked by D-test as described earlier (Yilmaz et al. 2007). Briefly, bacterial culture at 0.5 McFarland equivalents was inoculated onto a MHA plate, followed by placement of erythromycin (15 mg) and clindamycin (2 mg) disks at 15 mm apart. Plate was then incubated at 35 °C for overnight. A “D” shaped (blunted) clindamycin zone of inhibition towards erythromycin disc was interpreted as positive inducible clindamycin resistance.

### Statistical analysis

Numerical data was analyzed by Chi square test, odd ratio (OR) and 95 % confidence interval to determine statistical significance in the age, gender, infection origin (i.e. community vs. hospital) and antibiotic susceptibility pattern for MRSA and MSSA.

## Results

### Frequency of MRSA and MSSA

Out of total 280 *S. aureus* isolates, the frequency of MRSA and MSSA was 36.1 % (n = 101) and 63.9 % (n = 179) respectively. Both phenotypic (Cefoxitin disc and ORSAB) and Molecular (*mecA* detection) based MRSA screening methods showed similar results.

Specimen wise distribution showed that MRSA was most frequent in blood (48.7 %, OR 2.14), followed by fluids (41.7 %, OR 1.27), and pus (36.7 %, OR 1.04). MSSA was most frequent in throat specimens (82.6 %) followed by tissue samples (80 %). (Table 1).

**Table 1:** Prevalence of MRSA and MSSA isolated from Peshawar region of Khyber Pakhtunkhwa, Pakistan

Variables	*S. aureus* (N)	MRSA n (%)	MSSA n (%)	*X* ^2^	OR	95 % Confidence interval		*p* value
						Lower limit	Upper limit	
*Age* (*years*)								
1–9	66	25 (37.9)	41 (62.1)	0.122	1.10	0.6257	1.9593	0.727
10–19	20	7 (35)	13 (65)	0.011	0.95	0.3666	2.4663	0.918
20–29	40	19 (47.5)	21 (52.5)	2.643	1.74	0.8872	3.425	0.104
30–39	34	11 (32.3)	23 (67.7)	0.232	0.83	0.3862	1.7796	0.631
40–49	22	6 (27.3)	16 (72.7)	0.802	0.64	0.2435	1.7003	0.371
50–59	28	17 (60.7)	11 (39.3)	8.193	3.09	1.3855	6.8953	0.004
60–69	40	10 (25)	30 (75)	2.481	0.54	0.2548	1.169	0.115
70–79	30	6 (20)	24 (80)	3.763	0.40	0.1609	1.0342	0.052
	280	101 (36.1)	179 (63.9)					
*Sex*								
Male	182	62 (34)	120 (66)	0.906	0.78	0.4705	1.2984	0.341
Female	98	39 (39.8)	59 (60.2)					
*Specimen*								
Pus	120	44 (36.7)	76 (63.3)	0.032	1.04	0.6393	1.7119	0.857
Blood	82	40 (48.8)	42 (51.3)	8.122	2.14	1.2618	3.6258	0.004
Throat	46	8 (17.4)	38 (82.6)	8.329	0.32	0.1425	0.7147	0.004
Tissues	20	4 (20)	16 (80)	2.412	0.42	0.1365	1.2929	0.120
Fluids	12	5 (41.7)	7 (58.3)	0.170	1.27	0.3954	4.142	0.680
*Type*								
Community acquired	50	21 (42)	29 (58)	0.93	1.36	0.7277	2.5334	0.330
Hospital acquired	230	80 (34.8)	150 (65.2)					

*N* total numbers of *S. aureus* isolations; *X* ^*2*^ Chi square; *OR* Odd ratio

p value <0.05 is considered as statistically significant

The frequency of MRSA was found to be 39.8 and 34 % for female and male respectively. On the other hand, the frequency of MSSA in male and female was 66.6 and 60 % respectively (Table 1).

Methicillin-resistant *S. aureus* infection was higher among the age group 50–59 years (60.71 %, OR 3.09), followed by 20–29 years (47.5 %, OR 1.74), and 1–9 years (37.9 %, OR 1.10). MSSA infection was found higher among the age group 70–79 years (80 %), followed by 60–69 years (75 %) (Table 1).

Among MRSA, the frequency of community acquired MRSA and hospital acquired MRSA was 42 % (n = 21) and 34.8 % (n = 80) respectively. The frequency of community acquired MSSA and hospital acquired MSSA was 58 and 65.2 % respectively (Table 1).

### Antibiotic susceptibility pattern of MRSA and MSSA

The current study showed that all MRSA isolates were 100 % resistant to penicillin and cefoxitin; however entirely (100 %) susceptible to linezolid and vancomycin followed by rifampicin (81.2 %), chloramphenicol (77.2 %), clindamycin (75.2 %), minocycline (67.3 %) and cotrimoxazole (65.3 %) (Table 2). The data was further investigated for multi-drugs resistance (MDR) MRSA and extended drugs resistant (XDR) MRSA as per CLSI (2012), which showed that 15.84 % and 6.93 % MRSA were observed to be MDR and XDR respectively. D-test results revealed that 15.84 % (n = 16) of MRSA isolates were positive for inducible clindamycin resistance.

**Table 2:** Antibiotics resistance pattern of MRSA and MSSA isolated from Peshawar region of Khyber Pakhtunkhwa, Pakistan

Antimicrobial drugs	Drug susceptibility	MRSA n (%)	MSSA n (%)	p value
Penicillin (10 µg)	Susceptible	0 (0)	0 (0)	1
	Resistant	101 (100)	179 (100)	
Amikacin (30 µg)	Susceptible	52 (51.5)	179 (100)	<0.0001
	Resistant	49 (48.5)	0 (0)	
Chloramphenicol (30 µg)	Susceptible	78 (77.2)	179 (100)	<0.0001
	Resistant	23 (22.8)	0 (0)	
Ciprofloxacin (5 µg)	Susceptible	20 (19.8)	137 (76.5)	<0.0001
	Resistant	81 (80.2)	42 (23.7)	
Cefoxitin (30 µg)	Susceptible	0 (0)	179 (100)	<0.0001
	Resistant	101 (100)	0 (0)	
Erythromycin (15 µg)	Susceptible	01 (0.99)	54 (30.2)	<0.0001
	Resistant	100 (99.01)	125 (69.8)	
Gentamicin (10 µg)	Susceptible	44 (43.6)	167 (93.3)	<0.0001
	Resistant	57 (56.4)	12 (6.7)	
Clindamycin (2 µg)	Susceptible	76 (75.2)	177 (98.9)	<0.0001
	Resistant	25 (24.8)	2 (1.1)	
Minocycline (30 µg)	Susceptible	68 (67.3)	115 (64.3)	0.603
	Resistant	33 (32.7)	64 (35.7)	
Doxycycline (30 µg)	Susceptible	59 (58.4)	81 (45.3)	0.034
	Resistant	42 (41.6)	98 (54.7)	
Cotrimoxazole (1.25/23.75 µg)	Susceptible	67 (65.3)	167 (93.3)	<0.0001
	Resistant	34 (33.7)	12 (6.7)	
Linezolid (30 µg)	Susceptible	101 (100)	179 (100)	1
	Resistant	0 (0)	0 (0)	
Fusidic acid (10 µg)	Susceptible	60 (59.4)	148 (82.7)	<0.0001
	Resistant	41 (40.6)	31 (17.3)	
Rifampicin (5 µg)	Susceptible	82 (81.2)	179 (100)	<0.0001
	Resistant	19 (18.8)	0 (0)	
Vancomycin (30 µg)	Susceptible	101 (100)	179 (100)	1
	Resistant	0 (0)	0 (0)	
Moxifloxacin (5 µg)	Susceptible	15 (14.9)	174 (97.2)	<0.0001
	Resistant	86 (85.1)	5 (2.8)	

p value <0.05 is considered as statistically significant

MSSA showed high resistance (100 %) to penicillin followed by erythromycin (69.8 %) and doxycycline (54.7 %). In addition, MSSA showed 100 % sensitivity to amikacin, chloramphenicol, cefoxitin, linezolid, vancomycin and rifampicin (Table 2).

### MIC for vancomycin

As vancomycin is drug of choice against MRSA, therefore MIC of vancomycin was checked against MRSA isolates (n = 79) which revealed that 42 isolates had MIC 2 µg/ml followed by MIC 0.5 µg/ml (n = 12), 1 µg/ml (n = 12) 2.5 µg/ml (n = 6), 4 µg/ml (n = 6) and 8 µg/ml (n = 1). Overall 7 isolates showed vancomycin MICs of 4 or 8 µg/ml and thus were classified as VISA.

## Discussion

Methicillin-resistant *S. aureus* has been reported as a major bacterial pathogen involved in both community and hospitalized infections worldwide due to its increased virulence and continuous growing antibiotics resistance (Chambers and DeLeo 2009). In the present study we found 36.1 % frequency of MRSA among *S. aureus* isolates. A study conducted by Naeem et al. (2013) reported 31.5 % frequency of MRSA in Peshawar. There is continuous increase in the global prevalence of MRSA but generally frequency of MRSA in European countries is lesser as compared to other parts of the world. The reason may be the implementation of strict antibiotic prescribing policies and infection control practices in European countries (Askari et al. 2012).

In the present study MRSA was most frequent in blood (48.7 %); similar finding was also observed earlier (Hassan et al. 2014). No statistically significant differences were found regarding frequency of MRSA in gender or group of infection origin (hospital acquired vs community acquired). MRSA infection was higher among the age group 50–59 years (60.71 %), which is in line with another study conducted in Pakistan which reported high prevalence of MRSA in age group +44 years (Khan et al. 2014).

The multi-drug resistance in MRSA isolates has been a major problem worldwide which leads to ineffective therapy and increase in treatment expenses (Köck et al. 2010). The present study highlighted that high number of MRSA were multi-drug resistant which is in line with the previous findings which reported high rate of multi-drug resistance in MRSA (Tiwari et al. 2008; Khan et al. 2013).

We have found that all MRSA isolates tested in the present study were fully sensitive to vancomycin so it may be used as the drug of choice for treating multidrug-resistant MRSA infections. Similar findings were reported by various previous studies (Singh et al. 2013; Sipahi et al. 2013; Bhatt et al. 2014; Chelliah et al. 2014). In addition, all isolates were sensitive to linezolid which is in line with other studies (Sipahi et al. 2013; Vijayamohan and Nair 2014). Linezolid has been approved by FDA for treating MRSA infection, so linezolid could be a possible alternative of vancomycin (Micek 2007). Previous study showed that patient treated with linezolid had significantly higher survival rate than those treated with vancomycin (Wunderink et al. 2003). All MRSA isolates were resistant to cefoxitin which is in line with other studies (Wang et al. 2013; Ahmad et al. 2015). MRSA resistance to quinolones (ciprofloxacin) was also found to be very high (80.19 %) in the current study. This correlates with an earlier finding where it has been shown that MRSA was 86.59 % resistant to ciprofloxacin (Pandya et al. 2014).

The MIC profile revealed 7 (8.97 %) MRSA isolates as VISA. A previous study conducted at Pakistan also reported 13 % frequency of VISA using MIC by E test (Hakim et al. 2007). In the current study, we observed 15.84 % inducible clindamycin resistance in MRSA isolates which is in line with other studies (Yilmaz et al. 2007; Maninder et al. 2013). The high frequency of inducible clindamycin resistance in the present study and many other studies suggest that D-test should be adopted as routine practice in diagnostic microbiology to avoid clindamycin therapy failure (Maninder et al. 2013).

## Conclusion

The present study highlighted high proportion of MRSA and their multidrug resistance in clinical settings at Peshawar. The MDR MRSA is a potential threat to the public health at Peshawar as this bug can disseminate in community causing severe clinical conditions. Vancomycin and linezolid could be the drugs of choice in treating MRSA infection. Furthermore, MIC of vancomycin should be performed routinely to screen out VISA. In depth studies are needed in different geographical areas of the Pakistan to better understand the epidemiology and molecular mechanisms of drug resistance in MRSA.
